# Supplementary material for: DecoyFinder: Identification of Contaminants in Sets of Homologous RNA Sequences
Source: bioRxiv. 2024 Oct 15:2024.10.12.618037. Preprint. [Version 1] doi: 10.1101/2024.10.12.618037 (PMC11507696; doi:10.1101/2024.10.12.618037)
Supplement: Supplement 1 [file media-1.pdf]

Supplementary information to accompany:

DecoyFinder: Identification of Contaminants in Sets of Homologous RNA Sequences

Mingyi Zhu, Jeffrey Zuber, Zhen Tan, Gaurav Sharma, David H. Mathews

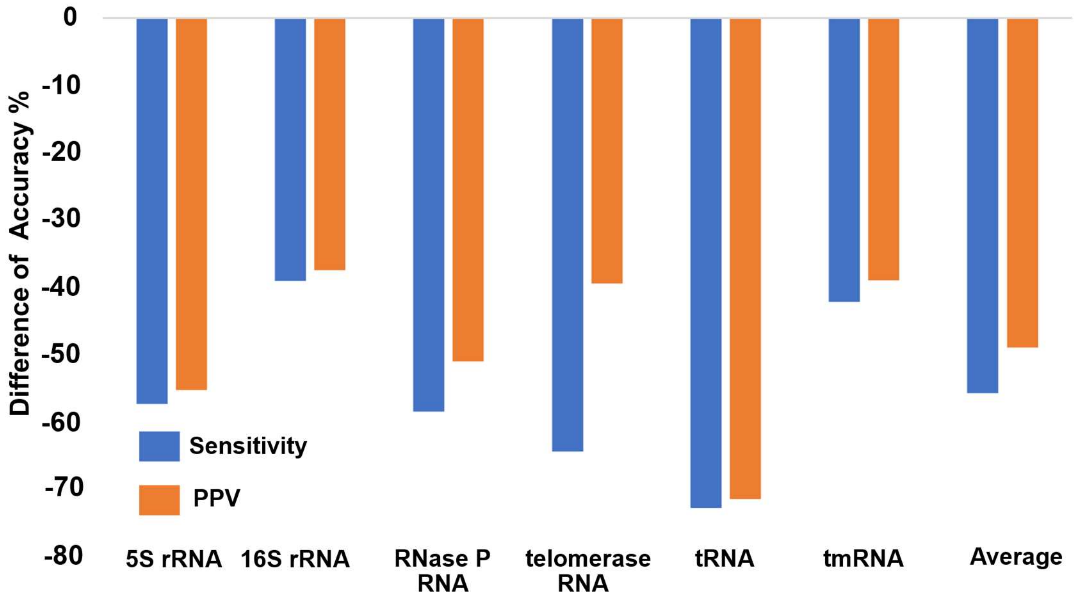

Supplementary Figure 1. Secondary structure prediction accuracy difference between shuffled decoy sequences and natural RNA sequences. Structures were predicted using maximum expected accuracy prediction (Lu, et al., 2009). For each RNA family, 100 sequences were chosen at random to generate a shuffled decoy. Sensitivity and PPV were calculated by comparing the predicted structure of shuffled decoy sequences with known structure of original sequence using the Scorer program in RNAstructure. The average change of sensitivity was -55.98% and the average change in PPV was -49.14%

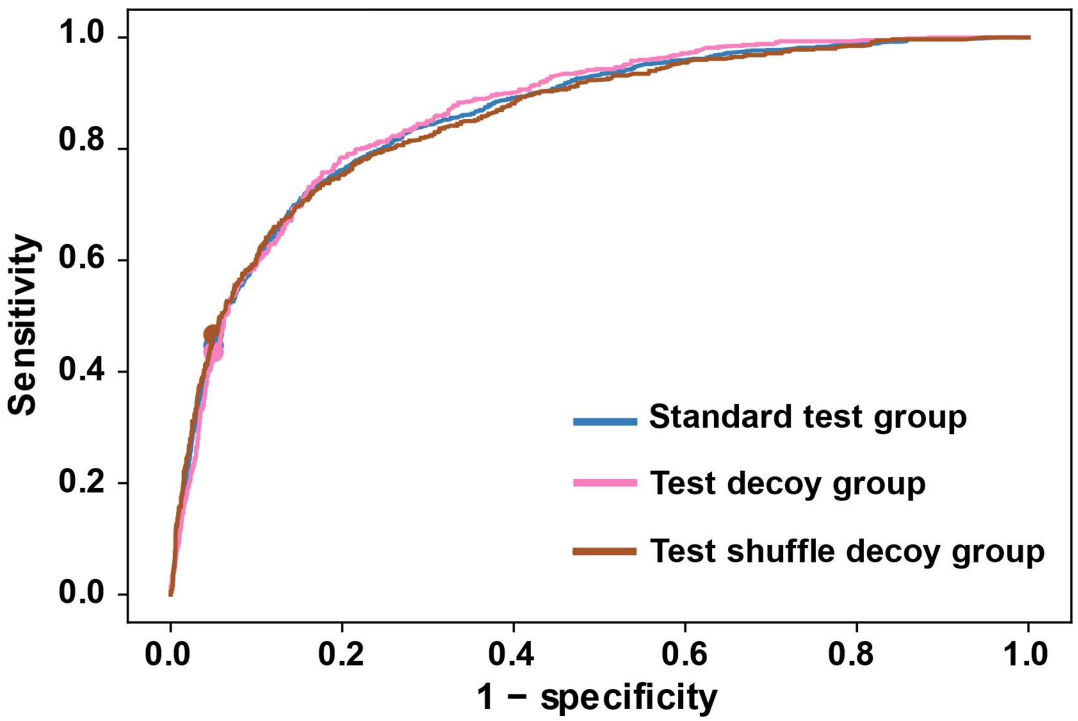

Supplementary Figure 2. ROC curves of the AdaBoost trained model for the standard test group with both types of decoys (blue), the test group with decoys from other families (pink), and the test group with shuffled decoys (brown). In both the decoy group and the shuffled decoy group, there is only one type of decoy.

Supplementary Table 1. The training set for the machine learning model. The Rfam families are 5S rRNA, internal ribosome entry site (IRES), malK-I RNA, RAGATH-18 RNA, RNase P RNA, skipping rope RNA, tRNA and U3 RNA. There are 900 sets of decoys from other families, 900 sets of shuffled decoys and 300 sets that do not include decoys. The sequences were randomly selected without replacement, therefore if a sequence has been chosen for one type of set (in a set of another family, shuffled decoy, or non-decoy) it will not be selected again. The selection of homologous and decoy families was random, but for each of the two types of decoys there are a total of 300 sets containing 1 decoy, 300 sets with 2 decoys and 300 sets with 3 decoys. The number of homologous sequences in all sets are randomly selected, ranging from 5 to 20.

Number of sets	Sequence family in set	Decoys in set	Decoy type (shuffle/other family/no decoy)	Sequence source	Sequences in set
13	5S rRNA	IRES	other family	Rfam	170
9	5S rRNA	RAGATH	other family	Rfam	139
11	5S rRNA	RNase P	other family	Rfam	168
14	5S rRNA	U3	other family	Rfam	212
11	5S rRNA	malK	other family	Rfam	180
14	5S rRNA	skipping rope RNA	other family	Rfam	196
29	5S rRNA	tRNA	other family	Rfam	417
11	IRES	5S	other family	Rfam	154
15	IRES	RAGATH	other family	Rfam	203
24	IRES	RNase P	other family	Rfam	323
18	IRES	U3	other family	Rfam	275
14	IRES	malK	other family	Rfam	198
17	IRES	skipping rope RNA	other family	Rfam	240
14	IRES	tRNA	other family	Rfam	161
20	RAGATH	5S	other family	Rfam	307
19	RAGATH	IRES	other family	Rfam	276
13	RAGATH	RNase P	other family	Rfam	197
13	RAGATH	U3	other family	Rfam	202
12	RAGATH	malK	other family	Rfam	190
14	RAGATH	skipping rope RNA	other family	Rfam	164
21	RAGATH	tRNA	other family	Rfam	287
13	RNase P	5S	other family	Rfam	184
18	RNase P	IRES	other family	Rfam	247
14	RNase P	RAGATH	other family	Rfam	201
15	RNase P	U3	other family	Rfam	198
15	RNase P	malK	other family	Rfam	250
11	RNase P	skipping rope RNA	other family	Rfam	166
16	RNase P	tRNA	other family	Rfam	218
15	U3	5S	other family	Rfam	206

15	U3	IRES	other family	Rfam	225
9	U3	RAGATH	other family	Rfam	122
14	U3	RNase P	other family	Rfam	198
25	U3	malK	other family	Rfam	347
18	U3	skipping rope RNA	other family	Rfam	279
22	U3	tRNA	other family	Rfam	318
16	malK	5S	other family	Rfam	217
17	malK	IRES	other family	Rfam	247
16	malK	RAGATH	other family	Rfam	265
18	malK	RNase P	other family	Rfam	249
24	malK	U3	other family	Rfam	317
12	malK	skipping rope RNA	other family	Rfam	184
13	malK	tRNA	other family	Rfam	195
25	skipping rope RNA	5S	other family	Rfam	373
8	skipping rope RNA	IRES	other family	Rfam	131
13	skipping rope RNA	RAGATH	other family	Rfam	195
15	skipping rope RNA	RNase P	other family	Rfam	202
21	skipping rope RNA	U3	other family	Rfam	287
24	skipping rope RNA	malK	other family	Rfam	338
9	skipping rope RNA	tRNA	other family	Rfam	108
18	tRNA	5S	other family	Rfam	262
19	tRNA	IRES	other family	Rfam	289
14	tRNA	RAGATH	other family	Rfam	212
19	tRNA	RNase P	other family	Rfam	234
14	tRNA	U3	other family	Rfam	192
22	tRNA	malK	other family	Rfam	326
17	tRNA	skipping rope RNA	other family	Rfam	229
170	5S rRNA	5S rRna	Shuffle	Rfam	2455
24	IRES	IRES	Shuffle	Rfam	349
189	RAGATH	RAGATH	Shuffle	Rfam	2797
29	RNase P	RNase P	Shuffle	Rfam	412
21	U3	U4	Shuffle	Rfam	316
82	malK	malK	Shuffle	Rfam	1123
204	skipping rope RNA	skipping rope RNA	Shuffle	Rfam	2825
181	tRNA	tRNA	Shuffle	Rfam	2670
52	5S rRNA	no decoy	no decoy	Rfam	691
8	IRES	no decoy	no decoy	Rfam	97
69	RAGATH	no decoy	no decoy	Rfam	888

9	RNase P	no decoy	no decoy	Rfam	126
7	U3	no decoy	no decoy	Rfam	89
28	malK	no decoy	no decoy	Rfam	324
57	skipping rope RNA	no decoy	no decoy	Rfam	656
70	tRNA	no decoy	no decoy	Rfam	950

Supplementary Table 2. The testing sets for DecoyFinder. The Rfam families are pemK RNA motif, small nucleolar RNA SNORD116, U4 spliceosomal RNA, U5 spliceosomal RNA and Y RNA. There are 300 sets of decoys from other families, 300 sets of shuffled decoys and 100 sets that contain no decoy. The sequences were randomly selected without replacement, therefore if a sequence has been chosen for one type of set (in a set of another family, shuffled decoy, or non-decoy) it will not be selected again. The selection of homologous and decoy families was random, but for each of the two decoy types there are a total of 100 sets containing 1 decoy, 100 sets with 2 decoys and 100 sets with 3 decoys. The number of homologous sequences in all sets are randomly selected, ranging from 5 to 20.

Number of sets	Sequence family in set	Decoys in set	Decoy type (shuffle/other family/non)	Sequence source	Sequence in set
18	SNORD116	Y RNA	other family	Rfam	214
18	SNORD117	U4	other family	Rfam	243
12	SNORD118	U5	other family	Rfam	132
11	SNORD119	pemK RNA motify	other family	Rfam	127
15	U4	Y RNA	other family	Rfam	217
15	U4	SNORD116	other family	Rfam	238
15	U4	U5	other family	Rfam	210
17	U4	pemK RNA motify	other family	Rfam	234
15	U5	Y RNA	other family	Rfam	231
15	U5	SNORD116	other family	Rfam	197
10	U5	U4	other family	Rfam	160
13	U5	pemK RNA motify	other family	Rfam	168
21	Y RNA	SNORD116	other family	Rfam	312
23	Y RNA	U4	other family	Rfam	333
13	Y RNA	U5	other family	Rfam	203
12	Y RNA	pemK RNA motify	other family	Rfam	172
18	pemK RNA motify	Y RNA	other family	Rfam	276
17	pemK RNA motify	SNORD116	other family	Rfam	252
11	pemK RNA motify	U4	other family	Rfam	150
11	pemK RNA motify	U5	other family	Rfam	160
14	SNORD116	SNORD117	Shuffle	Rfam	198
44	U4	U4	Shuffle	Rfam	614
41	U5	U5	Shuffle	Rfam	664
23	Y RNA	Y RNA	Shuffle	Rfam	372
178	pemK RNA	pemK RNA	Shuffle	Rfam	2597

	motify	motify			
4	SNORD116	non	non	Rfam	54
17	U4	non	non	Rfam	173
13	U5	non	non	Rfam	186
8	Y RNA	non	non	Rfam	105
58	pemK RNA motify	non	non	Rfam	769

Supplementary Table 3. The testing set for TurboFold's robustness with contamination present. The RNAStralign families used are 5S rRNA, 16S rRNA, RNase P RNA, telomerase RNA, tRNA and tmRNA. There are 700 sets of decoys taken from other families and 700 sets of shuffled decoys. Each set contains 4 homologous sequences and 1 decoy sequence. The sequences were randomly selected without replacement, therefore if a sequence has been chosen for one type of set (in a set of another family, shuffled decoy, or non-decoy) it will not be selected again.

Number of sets	Sequence family in set	Decoys in set	Decoy type (shuffle/other family/non)	Sequence source
15	5S rRNA	RNase P	other family	RNAStralign
18	5S rRNA	SRP	other family	RNAStralign
13	5S rRNA	16S rRNA	other family	RNAStralign
30	5S rRNA	tRNA	other family	RNAStralign
12	5S rRNA	telomerase RNA	other family	RNAStralign
12	5S rRNA	tmRNA	other family	RNAStralign
13	16S rRNA	RNase P	other family	RNAStralign
17	16S rRNA	SRP	other family	RNAStralign
20	16S rRNA	5S rRna	other family	RNAStralign
23	16S rRNA	tRNA	other family	RNAStralign
11	16S rRNA	telomerase RNA	other family	RNAStralign
16	16S rRNA	tmRNA	other family	RNAStralign
14	RNase P	16S rRNA	other family	RNAStralign
20	RNase P	5S rRNA	other family	RNAStralign
22	RNase P	SRP	other family	RNAStralign
9	RNase P	tRNA	other family	RNAStralign
20	RNase P	telomerase RNA	other family	RNAStralign
15	RNase P	tmRNA	other family	RNAStralign
18	SRP	16S rRNA	other family	RNAStralign
8	SRP	5S rRNA	other family	RNAStralign
13	SRP	RNase P	other family	RNAStralign
23	SRP	tRNA	other family	RNAStralign
23	SRP	telomerase RNA	other family	RNAStralign
15	SRP	tmRNA	other family	RNAStralign
16	telomerase RNA	16S rRNA	other family	RNAStralign
22	telomerase RNA	5S rRNA	other family	RNAStralign
16	telomerase RNA	RNase P	other family	RNAStralign
15	telomerase RNA	SRP	other family	RNAStralign
14	telomerase	tRNA	other family	RNAStralign

	RNA			
17	telomerase RNA	tmRNA	other family	RNAStralign
16	tmRNA	16S rRNA	other family	RNAStralign
17	tmRNA	5S rRNA	other family	RNAStralign
21	tmRNA	RNase P	other family	RNAStralign
9	tmRNA	SRP	other family	RNAStralign
18	tmRNA	tRNA	other family	RNAStralign
19	tmRNA	telomerase RNA	other family	RNAStralign
23	tRNA	16S rRNA	other family	RNAStralign
13	tRNA	5S rRNA	other family	RNAStralign
16	tRNA	RNase P	other family	RNAStralign
11	tRNA	SRP	other family	RNAStralign
17	tRNA	telomerase RNA	other family	RNAStralign
20	tRNA	tmRNA	other family	RNAStralign
100	16S rRNA	16S rRNA	Shuffle	RNAStralign
100	5S rRNA	5S rRNA	Shuffle	RNAStralign
100	RNase P	RNase P	Shuffle	RNAStralign
100	SRP	SRP	Shuffle	RNAStralign
100	telomerase RNA	telomerase RNA	Shuffle	RNAStralign
100	tmRNA	tmRNA	Shuffle	RNAStralign
100	tRNA	tRNA	Shuffle	RNAStralign

Supplementary Table 4. The RNA families' pairwise identity. The pairwise identity was determined by 30 randomly selected sequences from the TurboFold alignment.

Sequence family	Mean sequence identity	Usage
5S rRNA	56.9	Shuffle sequence accuracy test
16S rRNA	79.4	Shuffle sequence accuracy test
RNase P RNA	47.1	Shuffle sequence accuracy test
telomerase RNA	62.9	Shuffle sequence accuracy test
tRNA	50.6	Shuffle sequence accuracy test
tmRNA	49.7	Shuffle sequence accuracy test
5S rRNA	56.4	Model Training
internal ribosome entry site (IRES)	82.7	Model Training
malK-I RNA	58.6	Model Training
RAGATH-18 RNA	60.6	Model Training
RNase P RNA	47.9	Model Training
skipping rope RNA	53.8	Model Training
tRNA	42.2	Model Training
U3 RNA	65.9	Model Training
pemK RNA motif	65	Model Testing
small nucleolar RNA SNORD116	82.2	Model Testing
U4 spliceosomal RNA	59.8	Model Testing
U5 spliceosomal RNA	54	Model Testing
Y RNA	70.6	Model Testing

Supplementary Table 5. The RNase P RNA and RNase MRP RNA testing sets for DecoyFinder each contain 100 groups, with one group being homologous and the other being the decoy. The sequences were randomly selected without replacement; therefore, if a sequence has been chosen for one type of set (in a set of another family or non-decoy), it will not be selected again. The selection of homologous and decoy families was random, and each test set contains four homologous sequences and one decoy sequence from the other family.

Number of sets	Sequence family in set	Decoys in set	Decoy type (shuffle/other family/no decoy)	Sequence source	Sequences in set
100	RNase P	RNase MRP	other family	Rfam	500
100	RNase MRP	RNase P	other family	Rfam	500
